# Supplementary figures and images for: KMT2C knockout generates ASD-like behaviors in mice
Source: Front Cell Dev Biol. 2023 Jul 19;11:1227723. doi: 10.3389/fcell.2023.1227723 (PMC10394233; doi:10.3389/fcell.2023.1227723)

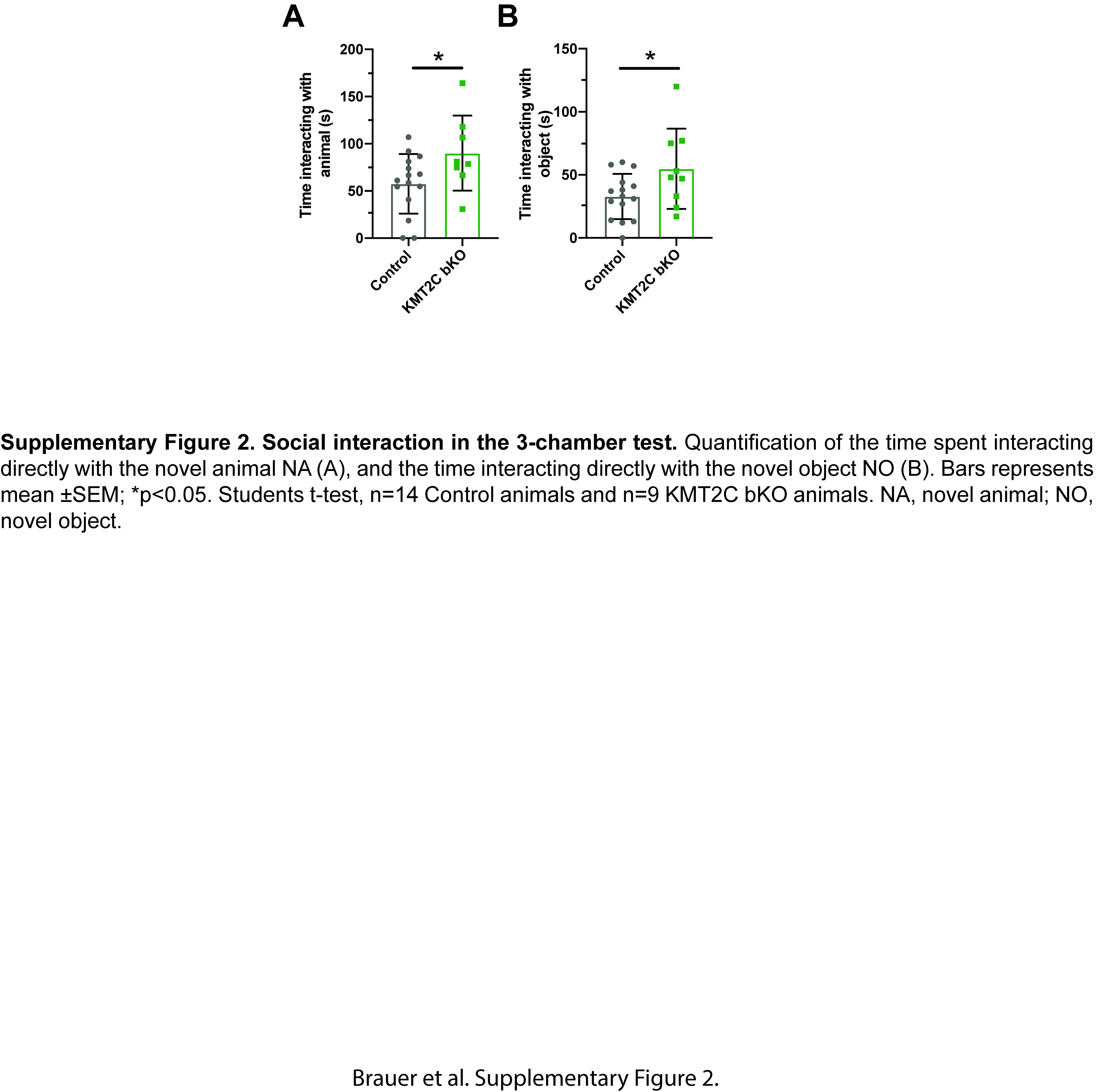

Supplement: Supplementary file 1 [file Image2.tif]

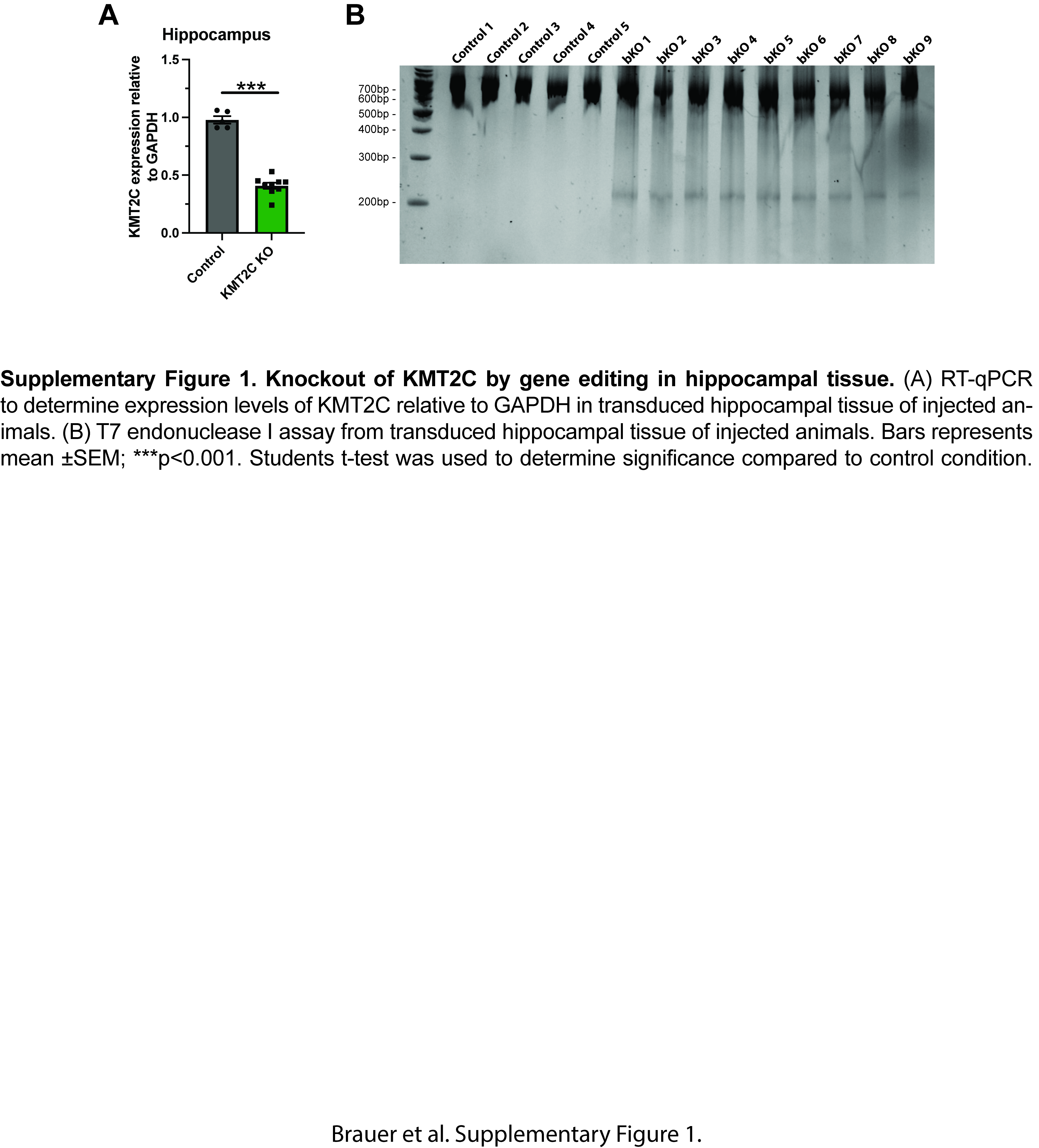

Supplement: Supplementary file 2 [file Image1.tif]
